# Comparing REM- and NREM-Related Obstructive Sleep Apnea in Jordan: A Cross-Sectional Study

**DOI:** 10.1155/2018/9270329

**Published:** 2018-08-12

**Authors:** K. Al Oweidat, S. A. AlRyalat, M. Al-Essa, N. Obeidat

**Affiliations:** ^1^Respiratory and Sleep Medicine, The University of Jordan, Amman, Jordan; ^2^Department of Ophthalmology, The University of Jordan, Amman, Jordan; ^3^Internal Medicine, The University of Jordan, Amman, Jordan

## Abstract

Obstructive sleep apnea (OSA) is a common disorder that includes an intermittent mechanical obstruction of the upper airway during sleep, which can occur either during rapid eye movement (REM) phase or non-REM (NREM) phase. In this study, we aim to evaluate the differences in demographic and polysomnographic features between REM- and NREM-related OSA in a Jordanian sample, using both the broad and the restricted definitions of REM-related OSA. All patients who were referred due to clinical suspicion of OSA and underwent sleep study were screened. We included patients with a diagnosis of OSA who had Apnea-Hypopnea Index (AHI) greater than or equal to five. We classified patients into REM-related OSA according to either the broad definition (AHI_REM_/AHI_NREM_ ≥ 2) or the strict definition (AHI_REM_ > 5 and AHI_NREM_ < 5 with a total REM sleep duration of at least 30 minutes), and patients with AHI_REM_/AHI_NREM_ less than two were classified as NREM-related OSA. A total of 478 patients were included in this study with a mean age of 55.3 years (±12.6). According to the broad definition of REM-related OSA, 86 (18%) of OSA patients were classified as having REM-related OSA compared to only 13 (2.7%) patients according to the strict definition. Significant differences were found between both NREM-related OSA and REM-related OSA according to the broad and to the strict definitions for arousal index (*p* < 0.001 and *p* < 0.032), respectively, duration of saturation below 90% (*p* < 0.001 for both), and saturation nadir (*p* < 0.036 and *p* < 0.013), respectively. No significant differences were found between this group and other OSA patients regarding age, BMI, ESS, and snoring. Our study showed that the stricter the definition for REM-related OSA, the milder the associated clinical changes.

## 1. Introduction

Sleep disordered breathing (SDB) spans a spectrum of disorders that includes obstructive sleep apnea (OSA), which implies an intermittent mechanical obstruction of the upper airway during sleep, leading to reduced airflow to the lungs [1]. Obstructive sleep apnea (OSA) is a common disorder affecting between 6% and 13% of the industrialized world [2, 3]. Daytime sleepiness, fatigue, snoring, early morning headache, and witnessed apnea are the most common presenting symptoms [4, 5]. Among the risk factors for OSA, obesity is the most important [6], as OSA is present in 40% of obese individuals and 70% of OSA patients are obese [7].

In patients with SDB, OSA events may occur throughout nonrapid eye movement (NREM) and rapid eye movement (REM) sleep. In REM sleep, hypotonia of upper airway muscles increases the risk for upper airway obstruction [8]. This hypothesis led to the emergence of the term “REM-related OSA” and increased the interest in studying this subtype of OSA. Although a lot of research was recently done on REM-related OSA, there is still no universally accepted definition for it, as a result of that, the prevalence of REM‐related OSA among the total number of patients with OSA undergoing polysomnography varies between 10% and 36% [9–12]. The most common criterion used in the literature for diagnosis of REM-related OSA is based on the ratio of Apnea-Hypopnea Index (AHI) during REM and NREM sleep (AHI_REM_/AHI_NREM_), where a value of two or more denotes REM‐related OSA. This definition is criticized for being broad, thus falsely including more patients under the category of REM‐related OSA. A more recent definition has emerged to restrict the criteria for diagnosing REM‐related OSA to having AHI_REM_ > 5 and AHI_NREM_ < 5 with at least 30 minutes duration of REM sleep [13]. Despite the findings of numerous studies concerning REM-related SDB, little is currently known about this type of SDB in Arab populations, since most reports have focused only on the features of REM-related SDB in Caucasian African American and recently Asian populations. In this study, we aim to evaluate the differences of demographic and polysomnographic features between REM‐ and NREM‐related OSA in a Jordanian sample, using both the broad and the restricted definitions of REM-related OSA.

## 2. Methods

This study was approved by the institutional review board (IRB) of the Jordan University Hospital, and it was conducted in accordance with the declaration of Helsinki latest update (2013).

### 2.1. Patients

During the period between June 2016 and April 2018, a total of 516 patients were referred to the sleep lab at the Jordan University Hospital; the indication for their referral was clinical suspicion of OSA suggested by symptoms such as snoring, increased daytime sleepiness, witnessed apnea, and early morning headache. Only patients who had an AHI greater than five were diagnosed to have OSA and were included in this study; accordingly, 38 patients were excluded.

### 2.2. Measurements

The overnight study consisted of continuous recordings of an electrocardiographic lead, right and left electrooculographic leads, submental, and two electroencephalographic leads. Respiration was monitored throughout the night with thermocouples at the nose and mouth, and with thoracic and abdominal strain gauges. Recording of the oxyhaemoglobin saturation (SaO_2_) and duration of saturation below 90% SpO_2_ (minutes) were obtained. The biophysiological changes on the polysomnography (PSG) device were evaluated using the 2.4 version of the American Academy of Sleep Medicine (AASM) Manual for the Scoring of Sleep and Associated Events [14]. Apnea was defined as a reduction in airflow by greater than 90% with a duration of at least 10 seconds in which there was a persistent respiratory effect. Hypopnea was defined as reduction of more than 30% in the airflow that was associated with an electroencephalographic arousal or a 3% or more drop in the SaO_2_. The Apnea-Hypopnea Index (AHI) was calculated as the total number of apneas and hypopneas per hour of total sleep time. Sleep state-dependent indices (i.e., NREM‐AHI and REM‐AHI) were also determined by dividing the number of events in NREM and REM sleep by the amount of NREM and REM time, respectively. Total snoring time was recorded throughout the study. The following demographic information was obtained: age, gender, and body mass index (BMI). Daytime sleepiness was assessed by a translated Arabic version of the Epworth Sleepiness Scale (ESS) completed by the patient himself/herself [15]. We validated the translated version of ESS before its use.

*Definitions*

  REM‐related OSA:
  The broad definition: AHI_REM_/AHI_NREM_ at least 2.
  The restricted definition: AHI_REM_ ≥ 5 and AHI_NREM_ <  5, with a total REM sleep duration of at least 30 minutes.
  NREM‐related OSA:
  AHI_REM_/AHI_NREM_ less than 2.

Comparison of patient's characteristics between the three groups, NREM‐related SDB and broad and strict types of REM‐related SDB, was done. We compared patient's demographics, snoring, desaturation time below 90%, nadir of O_2_%, and Epworth Sleepiness Scale (ESS).

### 2.3. Statistical Analysis

We used SPSS 21.0 (Chicago, USA) in our statistical analysis. We used mean (±standard deviation) to describe age, BMI, and other continuous variables. We used frequency (percentage) to describe gender, severity, and other nominal variables.

The independent sample *t*-test was used to compare the mean differences between REM‐related and NREM‐related OSA patient groups where we described the results in mean differences and a 95% confidence interval (95% CI). We used the chi-square test followed by *Z*-test for proportions (with Bonferroni correction) to assess gender differences in REM‐related and NREM‐related OSA patients and to study the difference in severity between REM-related and NREM-related OSA using the different definitions. A *p* value less than 0.05 was used as the significance threshold.

## 3. Results

A total of 478 patients were included in this study with a mean age of 55.3 years (±12.6), and there were 265 (55.4%) men and 213 (44.6%) women. Demographic (age, gender, and BMI) and polysomnographic (AHI severity, inclusion in either old or new definitions of REM-related OSA, ESS, arousal index, saturation below 90%, and saturation nadir) details of the included sample are shown in Table 1. According to AHI, 74 (15.5%) were classified as having mild OSA, 67 (14%) were moderate, and 337 (70.5%) were severe. Severity was significantly associated with gender (*p*=0.002), as 77% of men had severe OSA compared to 62.4% of women.  Table 2 details the characteristics for each grade of OSA.

According to the broad definition of REM‐related OSA, 86 (18%) of OSA patients were classified as having REM-related OSA compared to only 13 (2.7%) patients according to the strict definition. All patients who were classified as having REM-related OSA were included in the broad definition of REM-related OSA. 73 (15.3%) patients were REM‐related OSA included in the broader, but not in the strict definition. Upon comparing the severity of OSA between the broad, strict, and those satisfying the broad but not the strict, significant differences were found between REM‐related OSA and NREM‐related OSA, as shown in Table 3.

For patients with REM-related OSA according to the broad definition, the mean age was 52.2 years (±13.5). For gender differences, women had significantly (*p* < 0.001) higher percentage of REM‐related OSA (31%) compared to men (7.5%). Significant differences were found for arousal index (*p* < 0.001, mean difference between NREM‐related OSA (46.5 ± 21.9) and REM-related OSA (21.1 ± 14.5) of 25.4 with a 95% CI from 20.5 to 30.2), for duration of saturation below 90% (*p* < 0.001, mean difference between NREM-related OSA (35.4 ± 33.2) and REM-related OSA (18.2 ± 27.2) of 17.2 with a 95% CI from 9.7 to 24.8), and for saturation nadir (*p*=0.036, mean difference between NREM-related OSA (68.9 ± 16.3) and REM-related OSA (75 ± 14.1) of −6.1 with a 95% CI from −9.8 to −2.4). No significant differences were found between this group and other OSA patients regarding age, BMI, ESS, and snoring.

For patients with REM‐related OSA according to the strict definition, the mean age was 44.2 years (±14.1). For gender differences, women had significantly (*p*=0.003) higher percentage of REM‐related OSA (5.2%) compared to men (0.8%). Significant differences were found for arousal index (*p*=0.032, mean difference between NREM-related OSA (42.6 ± 22.7) and REM-related OSA (18.2 ± 14.4) of 24.4 with a 95% CI from 11.9 to 36.8), for duration of saturation below 90% (*p* < 0.001, mean difference between NREM‐related OSA (33.0 ± 33.0) and REM‐related OSA (8.6 ± 17.6) of 24.4 with a 95% CI from 6.4 to 42.4), and for saturation nadir (*p*=0.013, mean difference between NREM‐related OSA (69.6 ± 16) and REM‐related OSA (83.9 ± 7.5) of −14.1 with a 95% CI from −23.1 to −5.5). No significant differences were found between this group and other OSA patients regarding age, BMI, ESS, and snoring.

For REM-related OSA included in the broader but not in the strict definition, the mean age was 53.6 years (±13). For gender differences, women had significantly (*p* < 0.001) higher percentage of REM-related OSA (25.8%) compared to men (6.8%). Significant differences were found for arousal index (*p* < 0.001, mean difference between NREM‐related OSA (45.6 ± 22.2) and REM-related OSA (21.6 ± 14.5) of 23.9 with a 95% CI from 18.6 to 29.2) and for duration of saturation below 90% (*p*=0.001, mean difference between NREM‐related OSA (34.5 ± 33.1) and REM‐related OSA (19.9 ± 28.3) of 14.7 with a 95% CI from 6.6 to 22.8). No significant differences were found between this group and other OSA patients regarding age, BMI, ESS, snoring, or saturation nadir.

## 4. Discussion

In this study, we showed different characteristics for patients with REM-related OSA based on which definition is applied for diagnosis (broad versus strict). We found a milder nadir saturation reached and a lower duration of saturation below 90% when applying the strict definition of REM-related OSA compared to the broad definition. Moreover, the strict definition of REM‐related OSA yielded a lower arousal index compared to the broad definition. Upon comparing mean ESS and snoring duration, the mean ESS score or snoring duration for REM‐related OSA patients did not differ significantly from that for NREM‐related OSA patients. Finally, we showed frequency and characteristics of OSA and REM‐related OSA in Jordanians, a poorly studied population regarding this subject from the Middle Eastern ethnicity.

A previous large, community-based study on middle-aged adults did not find significant difference in daytime sleepiness on ESS and multiple sleep latency test (MSLT) between REM- and NREM-related OSA [16], a finding that is also appreciated in our study regardless of which REM-related OSA definition is used. This finding was also shown in a previous study where an increase in NREM AHI was found to be associated with daytime sleepiness as assessed by MSLT [17]. NREM-related OSA was also associated with poorer health-related outcomes, including mental health, compared to REM‐related OSA [16].

The stricter the definition of REM-related OSA, the lower the arousal index, and the milder the severity of OSA (according to AHI); these findings are also appreciated in a Japanese study that analyzed the features of REM‐related OSA according to the different definitions compared to NREM‐related OSA, and they found that REM‐related OSA is generally associated with lower arousal index along with milder severity of OSA.

In contrast to our study, they found a lower mean BMI among REM-related OSA patients compared to those with NREM-related OSA [18].

An earlier study that used the broad definition of REM-related OSA showed less disruption during sleep in REM-related OSA patients [16]. The use of saturation nadir was previously shown to correlate with disease outcome and was also demonstrated to possibly replace AHI in predicting OSA outcomes, including hypertension [19]. Duration of saturation below 90% was also studied and was found to correlate with OSA severity [20]. In our study, we investigated the effect of using broad and strict definitions of REM-related OSA on saturation nadir and duration of saturation below 90%, and we showed that the broad definition of REM-related OSA is associated with a higher mean difference in oxygen saturation nadir and a lower duration of saturation below 90% compared to NREM-related OSA.

The prevalence of OSA among middle-aged adults is estimated to be 2% in women and 4% in men in a large Canadian study [21]. A previous study in Jordan that used a Berlin questionnaire to screen for OSA showed that OSA is generally common among Jordanian population, with an overall higher risk in older men [22]. In our study that was conducted on Jordanian sample as well, we found an overall higher percentage of OSA among men. We also found that severer OSA is found mainly in NREM-related OSA and in older men who have higher BMI. The severer the OSA, the higher the arousal index and the ESS score, and the lower the saturation. However, snoring duration was not found to be associated with OSA severity.

This study has several limitations that should be considered in the future work. Common comorbidities, such as hypertension and diabetes, should be considered upon comparing the outcomes of patients with REM-related and NREM-related OSA. Moreover, majority of our sample had a severe form of OSA, which could raise a concern for a potential bias in representing OSA patients.

## 5. Conclusion

In this study, we found significant differences between both NREM-related OSA and REM-related OSA according to the broad and REM-related OSA according to the strict definitions for arousal index, duration of saturation below 90%, and saturation nadir, where REM-related OSA patients had milder findings. Generally, using the strict definition of REM-related OSA yields a milder OSA-related arousal index and a milder effect on oxygen saturation.

## Figures and Tables

**Table 1:** Demographic (age, gender, and BMI) and polysomnographic (AHI severity, inclusion in either old or new definitions of REM-related OSA, ESS, arousal index, saturation below 90%, and saturation nadir) details of the included sample.

	Mean	Standard deviation	Count	Percentage
Age	55	13		

Gender				
Male			265	55.4
Female			213	44.6

BMI	36.62	7.61		

AHI severity				
Mild			74	15.5
Moderate			67	14.0
Severe			337	70.5

Old definition				
No			392	82.0
Yes			86	18.0

New definition				
No			465	97.3
Yes			13	2.7

ESS	10.67	6.13		
Arousal index	41.92	22.89		
Saturation below 90%	32.31	32.85		
Saturation nadir	70.00	16.04		

AHI: Apnea-Hypopnea Index, BMI: body mass index, ESS: Epworth Sleepiness Scale.

**Table 2:** Comparing different severities of OSA (mild, moderate, and severe) regarding age, BMI (body mass index), ESS (Epworth Sleepiness Scale), arousal index, saturation nadir, and percentage snoring from total sleep.

	*p* value	Mean	Standard deviation	95% confidence interval	
				Lower bound	Upper bound
Age					
Mild	<0.001	49.42	13.8	46.23	52.61
Moderate		54.85	12.1	51.89	57.81
Severe		56.63	12.1	55.33	57.92
Total		55.26	12.6	54.13	56.40

BMI					
Mild	<0.001	33.31	7.6	31.55	35.07
Moderate		35.27	6.4	33.72	36.82
Severe		37.61	7.6	36.80	38.43
Total		36.62	7.6	35.93	37.30

ESS					
Mild	0.005	9.01	5.9	7.64	10.39
Moderate		9.61	6.0	8.14	11.08
Severe		11.25	6.1	10.59	11.90
Total		10.67	6.1	10.13	11.22

Arousal index					
Mild	<0.001	15.24	11.0	12.68	17.80
Moderate		23.33	11.9	20.42	26.23
Severe		51.48	19.3	49.41	53.55
Total		41.92	22.9	39.87	43.98

Saturation nadir					
Mild	<0.001	82.92	9.3	80.76	85.08
Moderate		79.13	8.5	77.06	81.21
Severe		65.34	16.0	63.63	67.06
Total		70.00	16.0	68.56	71.44

Duration of saturation below 90% (minutes)					
Mild	<0.001	10.01	21.01	5.14	14.88
Moderate		13.04	23.63	7.27	18.80
Severe		41.03	32.66	37.53	44.53
Total		32.31	32.85	29.35	35.26

% snoring					
Mild	0.342	21.64	21.4	16.67	26.61
Moderate		18.10	19.1	13.44	22.76
Severe		22.00	19.8	19.88	24.11
Total		21.39	19.9	19.60	23.19

**Table 3:** The difference in severity between REM-related OSA and NREM-related OSA according to different definitions of REM-related OSA. *p* value indicates the significance of difference between REM-related OSA and NREM-related OSA in each definition.

Definition	Type of OSA	OSA severity			Total number	*p* value
		Mild (%)	Moderate (%)	Severe (%)		
Broad	NREM-OSA	8.7	10.7	80.6	392	<0.001
	REM-OSA	46.5	29.1	24.4	86	

Strict	NREM-OSA	13.1	14.4	72.5	465	<0.001
	REM-OSA	100	0	0	13	

Broad without strict	NREM-OSA	11.6	10.4	78	405	<0.001
	REM-OSA	37	34.2	28.8	73	

## Data Availability

The data used to support the findings of this study are available from the corresponding author upon request.
